# Left-Handed Palming of the Needle Driver: A Technical Report for Surgical Trainees

**DOI:** 10.7759/cureus.57931

**Published:** 2024-04-09

**Authors:** Andrew Y Powers, Alex E Rosenthal, Charles E Mackel, Coleman P Riordan, Ziev B Moses

**Affiliations:** 1 Division of Neurosurgery, Department of Surgery, Beth Israel Deaconess Medical Center, Harvard Medical School, Boston, USA; 2 Department of Obstetrics and Gynecology, Beth Israel Deaconess Medical Center, Harvard Medical School, Boston, USA

**Keywords:** suturing, surgical technique, surgical ergonomics, medical education, equity

## Abstract

Left-handed surgical trainees are uniquely challenged when learning how to suture using standard needle drivers designed for right-handed individuals and often feel disadvantaged in comparison to their right-handed peers. “Palming,” a suturing technique that improves suturing mechanics and efficiency, cannot be achieved in the standard manner using the left hand. This paper proposes a previously undescribed technique for palming using the left hand that provides many of the same benefits as standard palming methods using the right hand, potentially reducing a common source of inequity in surgical training.

## Introduction

Suturing is one of the most fundamental technical skills learned in medical school. The subtleties of suturing are continually improved upon in residency, particularly in those with a surgical or procedural focus.

The standard method of holding a needle driver involves placing the first and fourth digits through the upper and lower halves of the needle driver, respectively. “Palming” is a suturing technique that involves holding the needle driver without one’s fingers in the openings of the instrument, improving the economy of movement when switching between the needle driver and other instruments, and increasing the needle driver’s rotational range through additional rotation with the fingers. Additionally, bringing the needle driver coaxial to forearm supination facilitates smooth needle passage, reducing tissue trauma and needle tip blunting [1].

A previous survey of orthopedic surgeons and trainees found a left-handed prevalence of 15% [2]. Left-handedness has often been considered an inconvenience and disadvantage in surgical training [3,4]. While left-handed cutting techniques with scissors have previously been described [5-8], palming the needle driver with the left hand has remained a poorly described ergonomic enigma and is a common source of inequity in surgical training [9,10].

## Technical report

**Figure 1:**
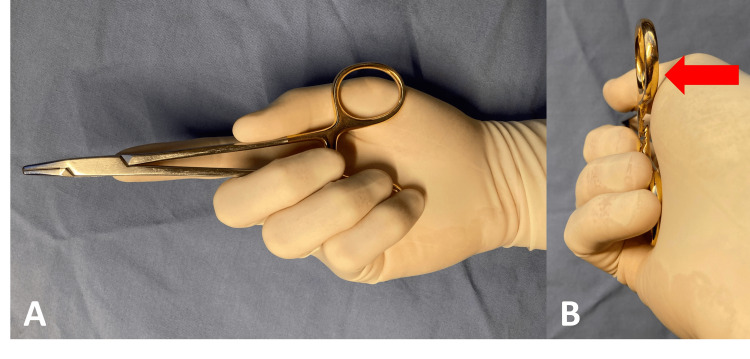
Standard technique for palming the needle driver in the right hand. (A) Side-on and (B) long-axis views of right-handed palming. The thenar eminence pushes the upper half of the needle driver (red arrow) to distract the locking mechanism and unlock the needle driver.

Unlocking a needle driver while palming with the right hand is accomplished by distracting the locking mechanism using pressure from the thenar eminence (Figure 1). Mirroring the standard right-handed palming grip and applying pressure with the left thenar eminence compresses, rather than opens, the locking mechanism [1]. Mirrored surgical instruments custom-designed for left-handed use or those with ambidextrous unlocking mechanisms [11] are expensive and seldom available to trainees [12,13].

This leaves left-handed surgeons with several imperfect options: palming with the right hand as described above, suturing using the left hand without palming, or mirroring the right-handed palming grip with the left hand to throw stitches but reverting to a non-palmed grip to unlock the needle driver. We describe an alternative left-hand grip that provides many of the same advantages as palming in the right hand.

**Figure 2:**
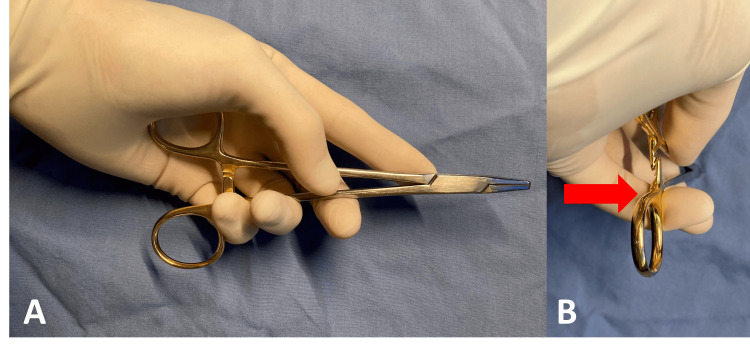
Proposed technique for palming the needle driver in the left hand. (A) Side-on and (B) long-axis views of an alternate left-handed palming technique. Digits three through five push the lower half of the needle driver (red arrow) to distract the locking mechanism and unlock the needle driver.

The upper opening of the needle driver is held in the center of the palm with the thumb around the needle driver’s upper half. The index finger provides stabilization distally, while digits three through five wrap around the outside of the lower half of the needle driver. In contrast to right-handed palming in which the thenar eminence is used to unlock the needle driver, digits three through five distract the locking mechanism to perform this function (Figure 2 and Video 1).

**Video 1:** Proposed technique for palming the needle driver in the left hand. The upper opening of the needle driver is held in the center of the palm. Digits three through five open the needle driver by distracting the locking mechanism. The needle driver can be rotated freely in the hand.

## Discussion

Left-handed trainees face several unique challenges when learning to operate due to their handedness, representing a significant source of inequity in surgical training. A previous survey of left-handed neurosurgical residents found that nearly half (43%) had difficulty using right-handed instruments, with a significant number (24%) feeling disadvantaged due to their handedness [12].

Historically, left-handed surgical trainees have adapted to the operating room environment through a variety of technical modifications. Many avoid the problem entirely by suturing with their right hand; however, this makes learning an unfamiliar task even more challenging for trainees. Further, it is not clear if using one’s non-dominant hand ultimately limits long-term technical proficiency. Alternatively, trainees may suture using the left hand without palming. While this approach grants the comfort of using one’s dominant hand, it forgoes the benefits of palming altogether. Lastly, some trainees will use a “hybrid” technique, mirroring the right-handed palming grip with the left hand to throw stitches but reverting to a non-palmed grip to unlock the needle driver. This method enjoys the mechanical benefits of palming without the same degree of efficiency.

While the technique proposed in this paper achieves the same efficiency benefits as right-handed palming, it does not bring the needle driver coaxial to the forearm. Thus, the range of rotation is increased through additional rotation with the fingers, but tissue trauma and ease of needle passage are not improved to the same degree as in right-handed palming. Left-handed surgeons may employ this technique either alone or as a faster unlocking method in conjunction with throwing stitches using the standard palming grip to obtain these mechanical benefits in exchange for some efficiency.

## Conclusions

Previously, left-handed surgical trainees have employed one of several adaptations to address the natural disadvantage they face when learning to suture using standard needle drivers that are designed for right-handed use. However, each of these methods has its own advantages and disadvantages. The grip modification reported in this paper provides a viable alternative suturing technique for left-handed surgeons that achieves many of the same mechanical and efficiency benefits that are enjoyed by their right-handed colleagues. This new technique has the potential to reduce a common source of inequity in surgical education.
